# Increase of somatic cell mutations in oxidative damage-sensitive drosophila

**DOI:** 10.1186/s41021-017-0090-z

**Published:** 2018-01-10

**Authors:** Ryota Koike, Tomoyo Uchiyama, Sakae Arimoto-Kobayashi, Keinosuke Okamoto, Tomoe Negishi

**Affiliations:** 1 Faculty of Pharmaceutical Sciences, Okayama University, Okayama, 700-8530 Japan; 2 Nihon Pharmaceutical University, Ina, Kita-Adachi-Gun, Saitama, 362-0806 Japan

**Keywords:** Uric acid, Somatic cell mutation, Drosophila, Oxidative stress

## Abstract

**Background:**

Oxidative damage is an important genotoxic source for almost all organisms. To efficiently detect mutations induced by oxidative damage, we previously developed a urate-null Drosophila strain. Using this Drosophila strain, we showed the mutagenic activity of environmental cigarette smoke (ECS) and the herbicide paraquat, which are known to produce reactive oxygen species (ROS). In the present study, we examined the mutagenic activities of carcinogenic mutagens that are considered to cause mutations by adduct formation, alkylation, or crosslinking of cellular DNA in the oxidative damage-sensitive Drosophila to evaluate how the oxidative damage induced by these mutagens is involved in causing mutations. In addition, we evaluated whether these oxidative damage-sensitive flies may be useful for mutation assays.

**Methods:**

We performed the wing-spot test in oxidative damage-sensitive Drosophila (urate-null strains) to examine the mutagenicity of 2-amino-3,8-dimethylimidazo[4,5-*f*]-quinoxaline (MeIQx), mitomycin C (MMC), 4-nitroquinoline N-oxide (4NQO), *N*-nitrosodimethyl-amine (NDMA), and *N*-nitrosodiethylamine (NDEA). We also observed the mutagenicity of X-ray irradiation as a control in which mutations should be mainly caused by oxidative damage.

**Results:**

As expected, the mutagenic activity of X-ray irradiation was higher in the urate-null Drosophila than in the wild-type Drosophila. The mutagenic activities of the tested compounds were also higher in the urate-null Drosophila than in the wild-type Drosophila. In experiments using another urate-null strain, the mutagenicity of *N*-nitrosodialkylamines was also higher in the urate-null flies than in the wild-type ones.

**Conclusions:**

The tested compounds in this study were more mutagenic in urate-null Drosophila than in wild-type Drosophila. It was supposed that ROS were generated and that the ROS might be involved in mutagenesis. The present results support the notion that in addition to causing DNA lesions via adduct formation, alkylation, or DNA crosslinking, these mutagens also cause mutations via ROS-induced DNA damage. As such, urate-null Drosophila appear to be useful for detecting the mutagenic activity of various mutagens, especially those that produce reactive oxygen. If the mutation rate increases on a mutation assay using urate-null Drosophila, it might suggest that the mutagen generates ROS, and that the produced ROS is involved in causing mutations.

## Background

We are constantly exposed, both exogenously and endogenously, to numerous factors that cause oxidative damage. It is well known that X-ray radiation and chemical oxidants exogenously induce reactive oxygen species (ROS) causing oxidative damage in organisms. ROS are also endogenously produced by biological reactions that take place in the mitochondria and microsomes (reviewed in [1]). Protection from ROS is indispensable for the wellbeing of organisms. Uric acid is chemically proven to be a powerful antioxidant and radical scavenger. It is likely an antioxidant in the human body, and human blood plasma contains approximately 300 μM of uric acid, a concentration even higher than that of ascorbic acid (around 50 μM) [2]. In other primates than human [3], birds [4], and some insects, such as silkworm [5] and Drosophila [6], it has been reported that uric acid plays roles as an antioxidant and as a radical scavenger. Muraoka and Miura reported that uric acid efficiently scavenged carbon-centered and peroxyl radicals that perform lipid peroxidation, especially under hydrophobic conditions [7]. ROS and radicals cause DNA damage that can lead to diseases, including cancer [8]. Previously, we reported that urate-null Drosophila mutants were sensitive to the toxicity of environmental cigarette smoke (ECS) [9, 10], and that the effects of the ECS were apparent even in the next generation [10]. We also reported that somatic cell mutations were induced by ECS and the herbicide paraquat in urate-null Drosophila*,* but not in wild-type Drosophila [11]. Many mutagenic carcinogens need to be activated to ultimate mutagens by metabolic enzymes, such as cytochrome 450 (CYP), and ROS are likely produced during this activation process. We postulated that a decrease of uric acid in test organisms might increase their sensitivity to the mutagenic activity of mutagens whose main mutagenic lesions have been considered other than oxidative damage. To determine whether oxidative damage is involved in the mutagenic process of typical carcinogenic mutagens, such as 2-amino-3,8-dimethylimidazo[4,5-*f*]-quinoxaline (MeIQx), mitomycin C (MMC), 4-nitroquinoline N-oxide (4NQO), and *N*-nitrosodialkylamines, we performed a somatic cell mutation assay using urate-null Drosophila.

## Methods

### Materials

*N*-nitrosodimethylamine (NDMA) and *N*-nitrosodiethylamine (NDEA) were purchased from Tokyo Kasei Co. Ltd. (Tokyo, Japan), MeIQx and 4NQO were purchased from Wako Pure Chemicals Industries, Ltd. (Osaka, Japan), and MMC was purchased from Sigma-Aldrich Inc. (Tokyo, Japan). These chemicals were used in aqueous solution, except for 4NQO, which was dissolved in an ethanol/tween 80 (2/1) solution.

### Drosophila strains

To detect somatic cell mutations, four urate-null Drosophila strains ([*y v ma-l; mwh*]*,* [*y v ma-l*]*,* [*ry*^*506*^]*,* and [*mwh, ry*^*506*^]) and two wild-type strains (Oregon-R and *ry*^*+*^) were used in the wing-spot test (see below). The Oregon-R and *y v ma-l* strains were kindly provided by Dr. H. Ryo (Osaka University, Suita, Japan). The *y v ma-l; mwh* strain was developed in our laboratory [11]. Both the *ry*^*506*^ and *mwh, ry*^*506*^ strains were obtained from the Drosophila Genomics and Genetic Resources Center at Kyoto Institute of Technology (Kyoto, Japan). The *y v ma-l* strain is deficient in xanthine dehydrogenase (XDH) activity due to the lack of a cofactor encoded in the *ma-l* (*maroon-like*) gene on the X chromosome [12]. The strain represented by *ry*^*506*^ is a mutant of the *rosy* gene on the third chromosome that is deficient in XDH activity due to a deletion in its gene [6]. In these urate-null mutants, the lack of uric acid and the accumulation of its precursors (xanthine and hypoxanthine) were confirmed by measuring the contents in the body fluid of both adult flies and larvae [9, 10]. The strain *ry*^*+*^ is speculated to be a revertant whose uric acid synthesis activity had recovered during breeding of *ry*^*506*^ in our laboratory (the uric acid content was 4.4 ± 1.1 nmol/mg protein, compared to 4.8 ± 0.8 nmol/mg protein in wild-type Oregon-R larvae). The presence of the *XDH* gene in *ry*^*+*^ flies was confirmed using PCR. The *multiple wing hairs* (*mwh*) gene encoded on the third chromosome is a marker gene that can be used to detect somatic cell mutations. These genotypes have been described by Lindsley and Zimm [13].

### Wing-spot test

**Fig. 1:**
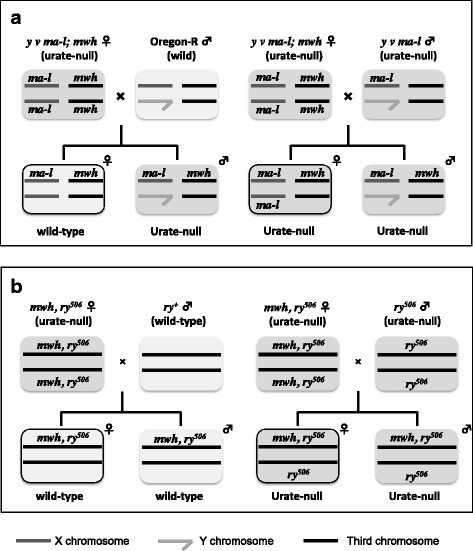
Mating schemes for preparing larvae for the wing-spot test with urate-null Drosophila. Urate-null mutants were prepared from *ma-l-*deficient flies (**a**), and from *ry*-deficient flies (**b**)

The Drosophila wing-spot test was performed to detect somatic cell mutations, including chromosomal recombination, according to the methods established by Graf et al. [14] with slight modifications [11]. To obtain third-instar larvae, virgin females of the genotype *y v ma-l; mwh* were crossed with males that were either wild type (Oregon-R) or *y v ma-l*. In another spot test, *mwh, ry*^*506*^ virgin females were crossed with *ry*^*506*^ or *ry*^*+*^ (wild-type at the *rosy* gene) males. Test compounds were supplied with the diet; this was prepared by dissolving a compound in 5 ml of distilled water before mixing it with 1.5 g of instant medium (Formula 4–24, Carolina Biological Supply, Burlington, NC). Third-instar larvae were placed on the medium containing the test compound, and kept at 25 °C until the individuals reached the adult stage. For observing the effects of X-ray irradiation (0.5 Gy/min), larvae were soaked in a 0.25 M sucrose solution on petri dishes (PD-47 50 ϕ × 11 mm; Advantic, Tokyo, Japan) with a hole for air exchange, and were irradiated with 3 or 10 Gy of X-ray, as described previously [15]. To observe the co-treatment effects of NDMA and uric acid, 126 mg of uric acid was mixed with 1.5 g of instant medium and 5 ml of 0.5 mM NDMA in aqueous solution. Somatic cell mutations were detected by counting the spots possessing mutant wing hairs under a microscope. The mutant spots were classified into small-single and large-single spots according to Graf et al. [14]. To avoid potentially confounding sex differences, only the wings from female flies were scored for urate-null flies (homozygotes; *ma-l*/*ma-l* and *ry*^*506*^/*ry*^*506*^) and urate-positive flies (heterozygotes; *ma-l*/+ and *ry*^*506*^/+), as shown in Fig. 1. The lack of uric acid and an increase in its precursors were confirmed in *mwh*, *ry*^*506*^/*ry*^*506*^ female flies using HPLC [16]; the content of uric acid in body fluid was 0.35 nmol/mg protein, that of hypoxanthine was 48.1 nmol/mg protein, and that of xanthine was 57.6 nmol/mg protein. Similarly, the lack of uric acid and an increase in its precursors has been confirmed in *ma-l*/*ma-l*; *mwh* female flies in a previous report [10]; the content of uric acid was 0.4 ± 0.3 nmol/mg protein, that of hypoxanthine was 43.2 ± 22.4 nmol/mg protein, and that of xanthine was 35.1 ± 11.7 nmol/mg protein. Statistical analysis of the wing-spot test results was performed using χ^2^ test described by Frei and Würgler [17] when total number of spots to be compared each other was one hundred and over, and using the tables shown by Kastenbaoum and Bowman [18] when total number of spots was under one hundred.

## Results

To examine the effects of uric acid deficiency on the mutagenic activity of various mutagens whose main mutagenic lesion is considered to be different from that due to oxidative damage, the wing-spot test was performed using urate-null Drosophila. We examined the activities of MeIQx, which causes the formation of DNA adducts that induce frame-shift mutations, MMC, which is a crosslinking agent that causes strand breaks, 4NQO, which causes the formation of DNA adducts that induce base substitution mutations, and *N*-nitrosodialkylamines, which alkylate DNA, inducing base substitutions. As positive control, we also examined the mutagenicity of X-ray irradiation, because X-ray is well known to induce mutation caused by mainly strand-break lesions via oxidative damage.

**Fig. 2:**
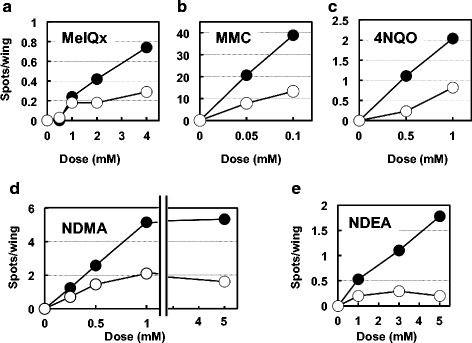
The mutagenicities of MIQx (**a**), MMC (**b**), 4NQO (**c**), NDMA (**d**), and NDEA (**e**) in either urate-null (closed circles) or wild-type (open circle) flies. Each spots-per-wing value represents the difference between the flies treated and not treated with the mutagen

The third-instar larvae from a cross between *y v ma-l, mwh* virgin females and *y v ma-l* or Oregon-R males were treated with the mutagens (Fig. 1a). The mutagenic activities of X-ray irradiation, MeIQx, MMC, 4NQO, NDMA, and NDEA are shown in Tables 1, 2, 3, 4, 5 and 6, respectively. In comparison to the wild-type flies, the rate of spontaneous mutations significantly increased in urate-null type flies, from 0.14 ± 0.04 to 0.44 ± 0.11 spots/wing on average, in all experiments of this study (Tables 1, 2, 3, 4, 5, 6 and 7). This increase was considered to be due to the production of ROS from regular endogenous biological reactions. As was expected, the mutagenicity of X-ray irradiation markedly increased in the urate-null flies, even at non-toxic doses (Table 1). The mutagenicity of each test compound also increased in urate-null flies when compared to that in wild-type flies at every tested dose. In Fig. 2, we plotted graphs with spots/wing from the data shown in Tables 2, 3, 4, 5 and 6, to make the difference between wild type strain and urate-null strain clear visually. Each value represents the difference between the flies treated and not treated with the mutagen. The mutagenic activities of all mutagens increased in the manner of linear dose-response, except for NDMA and NDEA at higher doses in the wild-type flies and NDMA at highest dose in urate-null flies.

**Table 1:** Mutagenicity of X-ray irradiation in urate-null mutants and wild-type flies

Dose (Gy)	Survival (%)	No. of wings	No. of spots (spots/wing)		
			Small	Large	Total
Exp. 1					
* y v ma-l; mwh* × Oregon-R F1 females (uric acid +)					
0	100	95	22 (0.23)	9 (0.09)	31 (0.33)
3	98	94	47 (0.50)	99 (1.05)^**^	146 (1.55)^*^
* y v ma-l; mwh* × *y v ma-l* F1 females (uric acid -)					
0	100	100	36 (0.36)	20 (0.20)	56 (0.56)
3	102	100	147 (1.47)^**^	173 (1.73)^**^	320 (3.20)^**^
Exp. 2					
* y v ma-l; mwh* × Oregon-R F1 females (uric acid +)					
0	100	100	15 (0.15)	5 (0.05)	20 (0.15)
10	112	102	277 (2.72)^**^	454 (4.45)^**^	731 (7.17)^**^
* y v ma-l; mwh* × *y v ma-l* F1 females (uric acid -)					
0	100	102	41 (0.40)	11 (0.11)	52 (0.51)
10	93	100	777 (7.77)^**^	1134 (11.3)^**^	1911 (19.1)^**^

^*^P ≤ 0.05, ^**^P ≤ 0.01, a significant increase in comparison to the corresponding control

**Table 2:** Mutagenicity of MeIQx in urate-null mutants and wild-type flies

Dose (mM)	Survival (%)	No. of wings	No. of spots (spots/wing)		
			Small	Large	Total
Exp. 1					
* y v ma-l; mwh* × Oregon-R F1 females (uric acid +)					
0	100	143	11 (0.08)	6 (0.04)	17 (0.12)
2	58	89	15 (0.19)^*^	5 (0.06)	20 (0.25)^*^
4	80	126	41 (0.33)^**^	11 (0.09)	52 (0.41)^**^
* y v ma-l; mwh* × *y v ma-l* F1 females (uric acid -)					
0	100	135	65 (0.48)	9 (0.07)	74 (0.55)
2	62	84	63 (0.75)^**^	13 (0.15)	76 (0.90)^**^
4	68	92	90 (0.98)^**^	29 (0.32)^**^	119 (1.29)^**^
Exp. 2					
* y v ma-l; mwh* × Oregon-R F1 females (uric acid +)					
0	100	151	19 (0.13)	4 (0.03)	23 (0.15)
0.5	68	92	11 (0.12)	6 (0.07)	17 (0.18)
1	67	108	31 (0.29)^**^	5 (0.05)	36 (0.33)^**^
2	57	94	28 (0.30)^**^	8 (0.09)	36 (0.38)^**^
* y v ma-l; mwh* × *y v ma-l* F1 females (uric acid -)					
0	100	117	46 (0.39)	11 (0.09)	57 (0.49)
0.5	88	105	42 (0.40)	9 (0.09)	51 (0.49)
1	95	111	66 (0.59)^*^	15 (0.14)	81 (0.73)^*^
2	50	53	48 (0.91)^**^	4 (0.08)	52 (0.98)^**^

^*^P ≤ 0.05, ^**^P ≤ 0.01, a significant increase in comparison to the corresponding control

**Table 3:** Mutagenicity of MMC in urate-null mutants and wild-type flies

Dose (mM)	Survival (%)	No. of wings	No. of spots (spots/wing)		
			Small	Large	Total
*y v ma-l; mwh* × Oregon-R F1 females (uric acid +)					
0	100	161	17 (0.11)	3 (0.02)	20 (0.12)
0.05	87	151	866 (5,74)^**^	349 (2.31)^**^	1215 (8.05)^**^
0.1	105	181	1816 (10.0)^**^	611 (3.38)^**^	2427 (13.4)^**^
*y v ma-l; mwh* × *y v ma-l* F1 females (uric acid -)					
0	100	113	65 (0.58)	10 (0.09)	75 (0.66)
0.05	44	50	769 (15.4)^**^	298 (5.96)^*^	1067 (21.3)^**^
0.1	56	64	2038 (31.8)^**^	488 (7.63)^**^	2529 (39.5)^**^

^*^P ≤ 0.05, ^**^P ≤ 0.01, a significant increase in comparison to the corresponding control

**Table 4:** Mutagenicity of 4NQO in urate-null mutants and wild-type flies

Dose (mM)	Survival (%)	No. of wings	No. of spot (spots/wing)		
			Small	Large	Total
*y v ma-l; mwh* × Oregon-R F1 females (uric acid +)					
0	100	100	10 (0.10)	3 (0.03)	13 (0.13)
0.5	102	100	21 (0.21)	25 (0.25)^*^	46 (0.46)^*^
1	96	100	42 (0.42)^*^	53 (0.53)^*^	95 (0.95)^*^
*y v ma-l; mwh* × *y v ma-l* F1 females (uric acid -)					
0	100	100	29 (0.29)	7 (0.07)	36 (0.36)
0.5	94	100	86 (0.86)^*^	61 (0.61)^*^	147 (1.47)^*^
1	61	100	108 (1.08)^*^	132 (1.32)^*^	240 (2.40)^*^

^*^P ≤ 0.01, a significant increase in comparison to the corresponding control

**Table 5:** Mutagenicity of NDMA in urate-null mutants and wild-type flies

Dose (mM)	Survival (%)	No. of wings	No. of spots (spots/wing)		
			Small	Large	Total
Exp. 1					
* y v ma-l; mwh* × Oregon-R F1 females (uric acid +)					
0	100	161	17 (0.11)	3 (0.02)	20 (0.12)
1	90	146	158 (1.08)^*^	79 (0.54)^*^	273 (1.62)^*^
5	14	22	32 (1.45)^*^	6 (0.27)^*^	38 (1.73)^*^
* y v ma-l; mwh* × *y v ma-l* F1 females (uric acid -)					
0	100	113	65 (0.58)	10 (0.09)	75 (0.66)
1	74	81	347 (4.28)^*^	57 (0.43)^*^	404 (4.99)^*^
5	10	10	58 (5.80)^*^	2 (0.20)	60 (6.00)^*^
Exp. 2					
* y v ma-l; mwh* × Oregon-R F1 females (uric acid +)					
0	100	140	17 (0.12)	2 (0.01)	19 (0.14)
0.25	91	166	98 (0.59)^*^	43 (0.26)^*^	141 (0.85)^*^
0.5	90	156	177 (1.13)^*^	71 (0.46)^*^	248 (1.59)^*^
1	88	184	373 (2.03)^*^	149 (0.81)^*^	522 (2.84)^*^
* y v ma-l; mwh* × *y v ma-l* F1 females (uric acid -)					
0	100	135	49 (0.36)	6 (0.04)	55 (0.41)
0.25	93	135	165 (1.22)^*^	56 (0.41)^*^	221 (1.64)^*^
0.5	98	143	340 (2.38)^*^	87 (0.61)^*^	427 (2.99)^*^
1	69	94	466 (4.96)^*^	136 (1.45)^*^	602 (6.40)^*^
Exp. 3					
* mwh, ry*^*506*^ × *ry*^*+*^ F1 females (uric acid +)					
0	100	82	110 (1.34)	13 (0.16)	123 (1.50)
0.5	89	85	298 (3.51)^*^	144 (1.69)^*^	442 (5.20)^*^
* mwh, ry*^*506*^ × *ry*^*506*^ F1 females (uric acid -)					
0	100	90	123 (1.37)	15 (0.17)	138 (1.53)
0.5	65	58	447 (7.71)^*^	137 (2.36)^*^	584 (10.1)^*^

^*^P ≤ 0.01, a significant increase in comparison to the corresponding control

**Table 6:** Mutagenicity of NDEA in urate-null mutants and wild-type flies

Dose (mM)	Survival (%)	No. of wings	No. of spots (spots/wing)		
			Small	Large	Total
Exp. 1					
* y v ma-l; mwh* × Oregon-R F1 females (uric acid +)					
0	100	140	17 (0.12)	2 (0.01)	19 (0.14)
1	92	164	29 (0.18)	6 (0.04)	35 (0.21)
3	90	158	38 (0.24)^*^	11 (0.07)	49 (0.31)^**^
5	66	114	41 (0.36)^**^	6 (0.05)	47 (0.41)^**^
* y v ma-l; mwh* × *y v ma-l* F1 females (uric acid -)					
0	100	135	49 (0.36)	6 (0.04)	55 (0.41)
1	120	169	111 (0.66)^**^	21 (0.12)^*^	132 (0.78)^**^
3	76	108	74 (0.69)^**^	25 (0.23)^**^	99 (0.92)^**^
5	30	44	57 (1.30)^**^	16 (0.36)^**^	73 (1.66)^**^
Exp. 2					
* y v ma-l; mwh* × Oregon-R F1 females (uric acid +)					
0	100	151	19 (0.13)	4 (0.03)	23 (0.15)
1	85	132	43 (0.33)^**^	19 (0.14)^**^	62 (0.47)^**^
3	72	103	43 (0.42)^**^	14 (0.14)^**^	57 (0.55)^**^
5	52	79	18 (0.23)	4 (0.05)	22 (0.28)
* y v ma-l; mwh* × *y v ma-l* F1 females (uric acid -)					
0	100	117	46 (0.39)	11 (0.09)	57 (0.49)
1	113	136	123 (0.90)^**^	36 (0.26)^**^	159 (1.17)^**^
3	105	125	209 (1.67)^**^	63 (0.50)^**^	272 (2.18)^**^
5	43	46	100 (2.17)^**^	29 (0.63)^**^	129 (2.80)^**^
Exp. 3					
* mwh, ry*^*506*^ × *ry*^*+*^ F1 females (uric acid +)					
0		92	128 (1.39)	11 (0.12)	139 (1.51)
1		62	118 (1.90)^**^	30 (0.48)^**^	148 (2.39)^**^
* mwh, ry*^*506*^ × *ry*^*506*^ F1 females (uric acid -)					
0		112	114 (1.31)	30 (0.34)	144 (1.66)
1		50	131 (3.12)^**^	57 (1.36)^**^	188 (4.48)^**^
Exp. 4					
* mwh, ry*^*506*^ × *ry*^*+*^ F1 females (uric acid +)					
0	100	82	110 (1.34)	13 (0.16)	123 (1.50)
1	87	88	135 (1.53)	28 (0.32)	163 (1.85)^*^
* mwh, ry*^*506*^ × *ry*^*506*^ F1 females (uric acid -)					
0	100	90	123 (1.37)	15 (0.17)	138 (1.53)
1	96	86	184 (2.14)^**^	51 (0.59)^**^	235 (2.73)^**^

^*^P ≤ 0.05, ^**^P ≤ 0.01, a significant increase in comparison to the corresponding control

**Table 7:** Mutagenicity of NDMA in the presence or absence of uric acid in the diet

NDMA (mM)	Addition of uric acid	Uric acid content in flies(nmol/mg protein)	No. of flies	No. of wings	No. of spots (spots/wing)		
					Small	Large	Total
*y v ma-l; mwh* × Oregon-R F1 females (uric acid +)							
0	–	256.6	79	100	9 (0.09)	2 (0.02)	11 (0.11)
	+	321.2	69	100	7 (0.07)	2 (0.02)	9 (0.09)
0.5	–	266.0	81	100	79 (0.79)	31 (0.31)	110 (1.10)
	+	371.1	72	100	38 (0.38)^§§^	13 (0.13)^§^	51 (0.51) ^§§^
*y v ma-l; mwh* × *y v ma-l* F1 females (uric acid -)							
0	–	0.08	68	100	23 (0.23)	6 (0.06)	56 (0.56)
	+	11.3	38	66	12 (0.18)	4 (0.06)	16 (0.24)
0.5	–	0.31	64	100	174 (1.74)	55 (0.55)	229 (2.29)
	+	11.1	32	54	88 (1.63)	24 (0.44)	112 (2.07)

^§^*P* < 0.05, ^§§^ *P* < 0.01, a significant decrease in the presence of uric acid in comparison to in the absence of uric acid

To confirm whether the increase in mutagenicity was dependent on a lack of uric acid, we performed the mutation assay using another available urate-null Drosophila strain, the *ry*^*506*^ strain. The third-instar larvae from a cross between *mwh, ry*^*506*^ virgin females and *ry*^*506*^ or *ry*^*+*^ males (Fig. 1b) were treated with NDMA or NDEA. As shown in Tables 4 and 5, the mutagenic activities of these mutagens were also higher in the urate-null Drosophila than in the wild-type flies. The mutation rates in the *ry*^*506*^ mutant were higher than that in the *ma-l* mutant both in the presence and in the absence of the mutagen. Unlike with the *ma-l* mutants, the spontaneous mutation rate was similar between *ry*^*506*^ and wild-type flies. It is unknown why the rate of spontaneous mutation did not rise in the *ry*^*506*^ mutants when compared to the wild-type flies.

When we examined the effects of the co-treatment with uric acid and NDMA in the diet, the addition of uric acid appeared to be toxic rather than protective in the urate-null mutants, and the mutagenic activity of NDMA could not be recovered. In contrast, in wild-type flies, the mutagenic activity of NDMA was suppressed in the presence of uric acid without any toxicity (Table 7).

## Discussion

The roles of uric acid as an antioxidant and radical scavenger have been widely recognized in vitro and in several organisms, including primates [1–6]. Previously, we reported that ECS and paraquat induced mutations in a urate-null mutant Drosophila strain, but not in wild-type Drosophila [11]. We showed that ROS were produced in the larval bodies exposed to ECS, which suggested that ROS are implicated in the mutagenicity of ECS.

In this study, we examined the effects of the lack of uric acid on the mutagenic activity of various mutagens. The main mutagenic lesions caused by the mutagens used are not considered to be those due to oxidative damage. Our results demonstrated that the mutagenic activity of all tested mutagens increased in urate-null mutants that had only trace levels of uric acid due to a deficiency in uric acid synthesis.

Xenobiotics incorporated into organisms are metabolized by the corresponding metabolic enzymes, and part of the metabolites can then become ultimate mutagens. In addition, such metabolic activation may produce several ROS from the reaction at CYP, and these ROS may also take part in mutagenesis [1].

MeIQx needs to be metabolized by CYP to be converted into an ultimate mutagen that can cause the formation of DNA adducts, thus causing mutations [19]. Murata et al. showed that a metabolite of MeIQx produced ROS in the presence of endogenous Cu(II) and induced oxidative DNA damage [20]. In addition, Singh et al. reported that uric acid inhibits the DNA breaking mediated by L-DOPA-Cu(II) [21]. The results of the present study support the notion that oxidative damage induced by the MeIQx metabolite may play a part in inducing mutations.

Although MMC is an anti-carcinogenic antibiotic that can cause the crosslinking of DNA, it has been reported that ROS are generated during its bioactivation, and that these ROS are correlated to cytotoxicity and are likely implicated in causing mutations [22, 23]. Our results are in line with these reports.

4NQO is converted to 4-hydroxyaminoquinoline N-oxide (4HAQO) via the reduction of a nitro group. 4HAQO is predicted to be a proximate mutagenic form that is implicated in DNA adduct formation [24]. 4HAQO is known to generate ROS [25], and 8-hydroxyguanine is detected in DNA from cultured cells treated with 4NQO [26]. In addition, gene expression that is correlated to the cellular redox condition could be altered by treatment with 4NOQ [27]. Therefore, in agreement with our data, oxidative damage produced by 4HAQO may be involved in mutagenesis and carcinogenesis, in addition to the adduct formation caused by 4NQO.

Evidence has accumulated that *N-*nitrosodialkylamines produce ROS via metabolic activation, that is, an increase in lipid peroxidation and suppression of oxidative stress by antioxidants have been reported [28–30]. NDMA and NDEA clearly showed increase of mutagenicity in both urate-null strains *ry*^*506*^ and *ma-l*. DNA alkylation has been considered to be the main mutagenic lesions of NDMA and NDEA; however, the ROS produced during metabolic activation also appear to be considerably implicated in mutagenesis and carcinogenesis. Moreover, it was suggested that the ROS involved in mutagenesis are decomposed by uric acid. Addition of uric acid to the medium did not recover the DNA-damaging effect of NDMA in urate-null mutants even when the uric acid concentration increased to 10 nmol/mg protein in body fluid. In contrast, in wild-type flies, the uric acid level increased to around 100 nmol/mg protein, and the DNA-damaging effect of NDMA seemed to recover partially. The reason why uric acid was toxic and ineffective in urate-null mutants remains to be investigated; however, in wild-type Drosophila*,* an increase in uric acid content might be effective for suppressing oxidative damage.

In this study, every mutagen tested showed increase of mutagenic activity in oxidative damage-sensitive Drosophila. It was considered that the uric acid content was sufficient in wild-type Drosophila to act as an endogenous antioxidant for the decomposition of ROS. Therefore, the mutagenic activity observed in wild-type Drosophila is considered to be mainly due to DNA lesions other than those caused by oxidative damage. The results of this study suggested that in addition to the mutagenesis due to adduct formation or the crosslinking of DNA, mutagenesis due to oxidative damage also occurs when the level of endogenous antioxidants decreased. Taken together, the urate-null Drosophila strains appear to be a useful tool for mutation assays. The spontaneous mutation rate was high in larvae from the crosses with the *ry*^*506*^ strain, and the fertility of the *ry*^*506*^ strain was comparatively lower than that of the *ma-l* strain. As such, the *ma-l* strain appears to be more suitable as a test organism in mutation assays.

## Conclusions

The compounds used in this study are thought to produce ROS during their bioactivation by metabolic enzymes, and these ROS might be involved in mutagenesis. All mutagens, including X-ray irradiation, caused higher rates of mutation in urate-null Drosophila than in wild-type Drosophila. It is well known that X-ray irradiation induces the production of ROS, which leads to mutations. Taken together, the results of this study support the notion that these mutagens cause mutations not only by causing the traditional lesions via adduct formation, alkylation or crosslinking, but also via the generation of ROS during bioactivation. As such, we propose that urate-null Drosophila may be useful for examining the mutagenic activity of various compounds, especially those that are thought to generate ROS. If the mutation rate increases on a mutation assay using urate-null Drosophila, it might suggest that the mutagen generates ROS, and that the produced ROS is involved in causing mutations.
